# Drying of Tarhana dough by Refractance Window™ technology under vacuum/atmospheric conditions: Characterization of physical and quality parameters

**DOI:** 10.1002/fsn3.3811

**Published:** 2023-11-06

**Authors:** Merve Seçil Bardakçi, Erkan Karacabey

**Affiliations:** ^1^ Department of Food Engineering Suleyman Demirel University Isparta Turkey

**Keywords:** bioactive compounds, Refractance Window™ drying, rheological behavior, tarhana, vacuum

## Abstract

This study aimed to produce dried tarhana using a refractance window drying (RWD) system. The drying process was also carried out under vacuum; the current study is the first in the literature. Using different heating mediums, the maximum temperature can be adjusted to a level above that used in RWD studies. Considering results, process time savings in RWD were over 85% and 75% compared to control groups (oven and hot air dryer), respectively. Tarhana samples dried in RWD were also faster (50%) under vacuum conditions than atmospheric ones. The highest preservation of total phenolic content (TPC) and total antioxidant activity (TAA) was for samples dried by RWD at 110°C under atmospheric conditions. Techno‐physical properties were better than the control group. The rheological behavior of tarhana soups was similar to pseudoplastic flow behavior and well defined by the Power law and Herschel–Bulkey models. In conclusion, RWD can be a promising technique for tarhana production.

## Graphical abstract

The results of the analyses showed RWD systems can be a promising technique for tarhana production under vacuum/atmospheric conditions. Process time savings in RWD were over 85% and 75% compared to control groups. Tarhana samples dried in RWD also faster (50%) under vacuum conditions. Quality properties were equal to or better than the control group in a much shorter time.

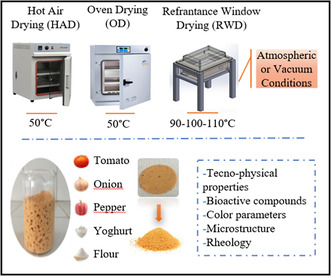

## 1 INTRODUCTION

Tarhana is a traditional fermented food product in different regional cuisines, from Anatolia to Eastern Europe and from Middle Eastern countries to North African ones. Tarhana has been called various other names in different regions. It is called “trahanas” in Greece, “tahonya” or “thanu” in Hungary, “tarhana” in Bulgaria, “talkuna” in Finland, “kishk” in Syria, Palestine, Jordan, Lebanon, and Egypt, and “kushuk” in Iraq and Iran (Daglioǧlu, 2000; Hayta et al., 2002; Koca et al., 2002). Cultural interactions with different civilizations have enabled a rich cuisine. Tarhana is consumed chiefly as soup. However, depending on the production and consumption habits of different regions, it can also be consumed as a snack, either wet or dried (Certel et al., 2007; Erbaş, 2003). Due to its ingredients, tarhana is a nutritious and easily digestible food product (Taşoğulları & Şimşek, 2020). Generally, tarhana is produced by mixing wheat flour, yogurt with yeast, various cooked vegetables, and spices (tomato, red pepper, onion, mint, salt, etc.). After the fermentation (via lactic acid bacteria [LAB] and yeasts), the mixture is dried, ground, and sieved. As a homemade production, it is traditionally dried under sunlight. Industrially, tarhana drying is carried out under hot air flow in a conventional oven. It is well known that convective hot air drying is the most common method used in the food industry (Daglioglu et al., 2002). However, depending on process conditions, undesirable losses may occur in physical properties (color, texture, etc.) and nutritional values of the dried product.

Today, due to increasing awareness, consumers are willing to buy high‐quality food. Thus, manufacturers aim to meet these expectations in addition to reducing production costs. Therefore, recently, drying techniques with significant advantages such as energy consumption, product quality, safety, environmental impact, dehydration cost, and efficiency have emerged and started to be applied in the food industry. One of them was patented by Magoon (Magoon, 1986) (Tacoma, WA), known as a Refractance Window™ Drying (RWD) technique.

RWD, also called conductive hydro drying, is a fourth‐generation drying technique (Ortiz‐Jerez et al., 2015). In the RWD system, the product to be dried (slice, puree, or juice) is spread on the plastic film. As reported by Nindo, Feng, Shen, Tang, and Kang (Nindo et al., 2003), 0.25‐mm‐thickness Mylar™ film (type D, DuPont, USA) was used during drying. Water is used as a heating medium for a typical RWD system. In the first opinion regarding the mechanism of the RWD system, the film is in contact with the heating medium at 95–97°C. When a wet material is spread over the film, a “window” for the product forms, through which heat and mass transfer occur (Baeghbali et al., 2010; Hernández‐Santos et al., 2016). As drying progresses and the moisture in the product structure decreases, the refractive index difference in the environment increases. Thus, it was stated that the window formed was closed as the thermal radiation of the water could no longer be transferred to the product. Baeghbali, Niakosari and Kiani (Baeghbali et al., 2010) stated that the RWD technique has three heat transfer mechanisms: conduction, convection, and radiation. However, Ortiz‐Jerez, Gulati, Datta, and Ochoa‐Martínez (Ortiz‐Jerez et al., 2015) revealed that most thermal energy is realized by conduction (99%). In this drying system, the moisture removed from the food is carried by airflow. Owing to this evaporative cooling in the RWD system, the temperature of the product rarely exceeds 70°C, even if the water temperature used is high (Nindo et al., 2007). Because of the lower product temperature, powders, flakes, and fruit pulp are produced with better physicochemical properties (Padhi & Dwivedi, 2022; Shende & Datta, 2019; Tontul et al., 2019).

Although RWD is a relatively new drying technique, it has become popular and started to be used in the drying of many different food materials, such as egg mixtures, milk, and dairy products (Tontul et al., 2021), probiotics (Yoha et al., 2021), and meat and meat products (Rostami et al., 2018), mainly due to the short drying time and high quality of the final product.

The drying technologies applied in the food industry have shown continuous improvements. Therefore, combined or integrated drying methods are frequently used in drying applications. Vacuum applications are integrated into the system, especially for heat‐sensitive food materials that can be damaged or changed when exposed to high temperatures (Montgomery et al., 1998; Péré & Rodier, 2002).

Because of the absence of air in the environment during vacuum drying, the oxidation reactions are reduced; thus, the quality characteristics of the processed samples are better (Erbay & Küçüköner, 2008). Vacuum dryers are facilitated, for example, for the drying of heat‐sensitive materials at low temperatures while at the same time preserving the characteristics of the product, such as taste, aroma, and rehydration ability (Montgomery et al., 1998; Péré & Rodier, 2002). Quintero Ruiz, Demarchi and Giner (Quintero Ruiz et al., 2014) reported that drying carried out under vacuum conditions is a suitable method to preserve the color of the product and the components such as ascorbic acid, phenolic compounds, anthocyanins, and lycopene.

In the present study, tarhana, a traditional fermented product, was dried using RWD. The system was adapted to operate under vacuum to understand its impact on the drying method, which is a new perspective for RWD technology. In addition, unlike typical RWD systems, heat transfer oil was used instead of water as the heating medium. Thus, the system can be used at temperatures above the corresponding process temperatures for RWD studies reported in the literature where water was used. The effects of the process parameters (temperature and pressure) on the process efficiency were investigated and compared with those of the control group. In addition, the quality of the dried product was evaluated in terms of physico‐chemical properties (color, phenolics, and antioxidant activity). This study is essential because there are no reports of RWD performed under vacuum conditions or of tarhana being dried by RWD. In this respect, the present study is the first of its kind in literature.

## 2 MATERIALS AND METHODS

### 2.1 Tarhana production

Tarhana dough was prepared according to the method of Ibanoglu, Ainsworth, Wilson and Hayes (Ibanoglu et al., 1995). Onions (60 g) were chopped and homogenized with 50 mL of distilled water using a hand blender (MR 404 Braun, Germany) for 30 s. Tomato paste (60 g), salt (40 g), red pepper (10 g), and mint (1 g) were manually mixed for 30 s. The mixture was boiled for 10 min. Yogurt (250 g), wheat flour (500 g), and yeast (10 g) were added to the mixture, which was cooled to room temperature after boiling and kneaded by hand. The resulting dough was transferred into plastic containers and incubated at 30°C for 48 h in an incubator, after which it was stored at 4°C until drying experiments.

To achieve uniform thickness, tarhana dough was passed through a dough sheeter (Penguin PNG‐2000, Turkey) with different thickness adjustment levels. To prevent the dough from sticking, first of all, ten grams of wheat flour were added to a hundred‐gram dough, and then the final mixture was rolled to a thickness of 3 mm. Fifty millimeters of tarhana discs were cut using a specially produced knife. Discs were spread on a mylar film surface and then subjected to the drying process.

### 2.2 Drying experiments

**FIGURE 1:**
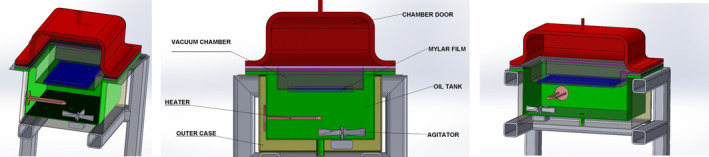
Schematic diagram of the pilot‐scale RWD system from different viewpoints.

RWD was conducted using a pilot‐scale dryer with a vacuum chamber (Figure 1) (Eraktek Machine, Konya, Turkiye). In this system, for heat transfer, oil 32 (Petrol Office, Turkiye) was used as a heating medium instead of water to make the system available to work at temperature levels above 100°C. The two sections in the RWD system are separated from each other with Mylar film (0.25 mm thickness) having high infrared radiation transmittance. The bottom section is an oil bath, storing heat‐transfer oil. The section above the Mylar film is removable and operated according to pressure conditions. If a vacuum is applied, the top section is closed on Mylar film without any leakage, and a closed vacuum chamber is formed. At atmospheric pressure, that top section is not used, and drying is carried out while the top surface of the Mylar film is open to the atmosphere. In the drying equipment, the temperature inside the oil bath is controlled. The food materials subject to the study were dried by spreading on the film. In the drying process under vacuum conditions, the system pressure applied to the drying cabinet was adjusted to 560 mm Hg.

For drying, approximately 60 gof tarhana discs were arranged as a single layer on the plastic film. Tarhana samples were dried at 90, 100, and 110°C under vacuum or atmospheric conditions. Control groups were also considered for comparison of the RWD system with traditional ones. Two control groups that differed based on the technologies used were studied. For the first one, tarhana discs were dried in a hot air dryer at 50°C (HAD) (Microtest, MKD, Turkey) under an air flow rate of 1.3 ± 0.02 m.s^−1^ (flowing parallel to the drying surface tray). The second control group was dried in an oven dryer (OD) without air flow at 50°C (Nüve, FN500, Türkiye). The initial moisture content of approximately 39% tarhana dough was dried until the final moisture content was reduced below 10% (wet basis, wb), which was reported in TSE (TSE, 2004). After drying, tarhana discs were grounded (Sinbo, Scm 2934) and sieved to achieve a particle size of tarhana powder less than 400 μm. Powder samples were packaged under vacuum (Vacuum Packaging Machine, DZ‐350/MS, CHINA) in a polyamide/polyethylene bag and stored at −45°C until analyses.

### 2.3 Drying characteristics

#### 2.3.1 Moisture content and water activity

Tarhana powder's moisture content (MC) was determined using a moisture analyzer (DBS 60–3, Kern & Sohn GmbH, Germany). Three grams of sample were weighed, and moisture content was measured at 105°C with an accuracy of 0.01 g. The samples' water activity (aw) was determined using a water activity meter (Thermoconstanter TH 200, Novasina, Axair Ltd., Switzerland) at 25 ± 2°C. Experiments were performed in triplicate, and the values were expressed as mean ± SE.

#### 2.3.2 Determination of acidity degree, pH values, and ash content

To determine the pH value of tarhana powders, approximately 10 g of sample was mixed with 100 mL of distilled water and filtered through filter paper (AACC, 2000). A pH meter probe was immersed in the obtained filtrate at room temperature. The pH value was measured with a pH meter (S210, Metler Toledo, Switzerland).

The acidity degree of dried powdered tarhana was determined according to the Tarhana Standard (TS 2282) (TSE, 2004). For this purpose, 10 g of tarhana sample was weighed, 50 mL of neutralized 67% ethyl alcohol was added, and then homogenized for 5 min. The mixture was then filtered through filter paper. 10 mL was taken from the filtrate, and 2–3 drops of phenolphthalein indicator were added and titrated with a 0.1 N sodium hydroxide solution until a stable pink color was formed. The acidity degree of the tarhana samples was determined by multiplying the amount of spent sodium hydroxide solution by 5.

The total ash content of tarhana powder was determined according to AACC (AACC, 1990). Almost 3–5 g of tarhana powder was weighed in porcelain crucibles and burned gradually in an ash oven (Nüve MF 120, Türkiye) at 550°C. The burning process continued until the ash's color turned white. The cooled crucibles were weighed again, and the percentage of ash was calculated. The results were given as g ash/100 g dry matter.

#### 2.3.3 Total phenolic content and total antioxidant activity

To determine the total phenolic content (TPC) and total antioxidant activity (TAA) of dried tarhana, the extraction process was carried out, as reported by Akan and Ocak (Akan & Ocak, 2019). Approximately 5 g of powder was weighed, and 50 mL of methanol was added and homogenized with Ultra‐Turrax (T‐18, IKA, Germany) for 2 mins. The mixture was extracted for 1 h at 40°C, and then the sample was centrifuged (Nüve NF 800R, Türkiye) to separate the supernatant. The extraction process was carried out in two parallels.

The method suggested by Singleton and Rossi (Singleton & Rossi, 1965) was used to determine the TPC of tarhana. Forty micro liters of the prepared extract were taken, and 2.4 mL of distilled water was added and vortexed. Then, 0.2 mL of Folin–Ciocalteu solution was added and vortexed once more. Following this, the incubation period was started by adding 0.6 mL of saturated sodium carbonate (Na_2_CO_3_). 0.76 mL of distilled water was added to the resulting mixture and incubated in the dark for 2 h. Sample absorbance was measured with a spectrophotometer (T70 + UV/VIS spectrophotometer, PG Instruments, UK) at 765 nm wavelength. The result was given in terms of “mg gallic acid/100 g dry matter”. The TPC of the sample was determined as the mean of two parallels.

The ABTS method reported by Re, Pellegrini, Proteggente, Pannala, Yang, and Rice‐Evans (Re et al., 1999) was modified and used to determine the TAA of the sample. ABTS/water solution was adjusted to 7 mM. ABTS radical cation (ABTS^•+^) was produced by reacting ABTS stock solution with 2.45 mM potassium persulfate (final concentration) and allowing the mixture to stand in the dark at room temperature for 12–16 h. The ABTS solution was diluted with ethanol to achieve an absorbance of 0.700 (±0.02) at 734 nm. 10 μL of the prepared extract and 990 μL of ABTS solution were mixed. The absorbance value (at 734 nm) was read twice, at the beginning and 6 min later. The result was calculated as mg TEAC (Trolox equivalent antioxidant capacity)/100 g dry matter. The TAA of the sample was determined as the mean of two parallels.

#### 2.3.4 Color measurement

A colorimeter (3nh Focus on Color, NH310 High‐Quality Portable Colorimeter, Shenzhen 3NH Technology CO., LTD.) was used to determine the color values of tarhana powder. The measurement was carried out based on the CIE Color Scale (L*, a* and b*) method. Readings were noted at five different points of spread tarhana powder for each sample, and the result was given as a mean of those readings for each color parameter. The total color change (ΔE) value was calculated by using L*, a*, b*, C*, and h° values to determine the color change occurring in the dried samples with respect to reference (Equation (1)). While calculating the total color change, the control sample of HAD was used as a reference for tarhana powder.

(1) $$∆E=\sqrt{{\left(L^{*}-L_{0}^{*}\right)}^{2}+{\left(a^{*}-a_{0}^{*}\right)}^{2}+{\left(b^{*}-b_{0}^{*}\right)}^{2}}$$

#### 2.3.5 Viscosity

The viscosity of the tarhana–water mixture was measured with a Brookfield (Middleboro, Massachusetts, USA) viscometer. To determine the viscosity value, the method of Steffe (Steffe, 1996) was used, and a tarhana–water mixture was prepared by mixing 10 g of tarhana powder with 90 mL of water. This mixture was boiled and then stirred for 8 min. During cooling, the viscosity value of the sample was measured at nine different speeds (5, 10, 20, 30, 50, 60, 100, 150, and 200 rpm) at successive three different temperature levels (40, 55, and 70°C).

Newtonian, Power law, Herschel–Bulkley, and Casson were used to model the rheological behavior of tarhana powders. The model equations are given below.

(2) $$\text{Newtonian Model}:\tau =k\left(\gamma \right)$$

(3) $$\text{Power}\;\mathrm{law}\;\text{model}:\tau =k{\left(\gamma \right)}^{n}$$

(4) $$\text{Herschel}–\text{Bulkey model}:\tau =\tau_{0}+k{\left(\gamma \right)}^{n}$$

(5) $$\text{Casson Model}:\tau^{0,5}={\tau_{0}}^{0,5}+k{\left(\gamma \right)}^{0,5}$$

where *τ* represents apparent viscosity, *k* represents the consistency coefficient, *γ* represents shear rate, and *n* represents the flow behavior index.

#### 2.3.6 Determination of powder properties

Techno‐physical properties, including solubility, hygroscopicity (%), foam capacity and stability, water and oil holding capacity, porosity, flowability [Carr index (CI) (Carr, 1965)], and cohesiveness [Hausner ratio (HR) (Hausner, 1967)] values, of tarhana powder were determined according to the procedures described by Dailami (Dailami, 2009), Santhalakshmy, Bosco, Francis and Sabeena (Santhalakshmy et al., 2015), Ng and Sulaiman (Ng & Sulaiman, 2018), Hayta, Alpaslan and Baysar (Hayta et al., 2002), and Jinapong, Suphantharika and Jamnong (Jinapong et al., 2008), respectively. For powder flowability, the following table was used to classify tarhana samples (Table 1).

**TABLE 1:** Classification of powder flowability based on Carr Index (Carr, 1965) and Hausner Ratio (Hausner, 1967).

Parameters	Values	Flowability cohesiveness
Carr index (%)	<15	Very good
	15–20	Good
	20–35	Fair
	35–45	Bad
	>45	Very bad
Hausner ratio	<1.2	Low
	1.2–1.4	Intermediate
	>1.4	High

#### 2.3.7 Scanning electron microscopy

To observe structural changes due to the drying processes under atmospheric conditions or under vacuum, SEM images of tarhana powder were taken using scanning electron microscope (FEI Quanta FEG 250, Japan). SEM images of fixed powders were taken in low vacuum mode at 60 Pa without a conductive coating, using a gaseous secondary electron detector for the environmental SEM chamber.

#### 2.3.8 Statistical analysis

A Duncan multiple comparison (*p* ≤ .05) test was used to determine the statistical difference between the means. Statistical analyses were performed with the Minitab Statistical Package Program (version 16.2.3.0) (Minitab Inc.). Results are given as mean ± SE.

## 3 RESULTS AND DISCUSSION

### 3.1 Drying times

In the food industry, minimizing the drying time of the dried product is of great importance not only for the quality of the final product but also for the economy. The drying time of tarhana produced with the RWD system is given in Table 2. It decreased with a temperature increase and under vacuum conditions in the RWD system (*p* ≤ .05). Tarhana dried under vacuum conditions in shorter time values, which were reduced by 49.84%, 51.97%, and 53.07% compared to drying under atmospheric conditions at 90, 100, and 110°C, respectively. Saving on process time was more than 85% and 75% compared to OD and HAD, respectively. As a result, the RWD system may be considered a promising method for tarhana drying because it reduces process time while maintaining product quality thanks to functional characteristics like TPC and TAC that are identical to the control ones. Additionally, the authors suggest that reaching a definite judgment on the energy consumption and cost performance of the RWD system is crucial to achieving better system evaluation. Thus, it is thought that detailed analyses are required to this extent.

**TABLE 2:** Drying times, moisture content, water activity, pH, acidity level, and ash content results of tarhana powders dried with different drying methods.

Drying process	Temperature (°C)	Drying time (minimum)	Moisture content (%)	Water activity (a_w_)	pH	Acidity degree	Ash content^a^ (%)
A‐RWD	90	125.80 ± 0.93^c^	8.64 ± 0.13^a,b^	0.493 ± 0.001^a^	4.78 ± 0.00^d^	14.58 ± 0.03^b^	7.53 ± 0.01^d^
	100	76.00 ± 1.48^d^	8.80 ± 0.17^a^	0.476 ± 0.003^b^	4.80 ± 0.00^c,d^	12.40 ± 0.15^d,e^	7.57 ± .0.3^c,d^
	110	52.00 ± 0.78^f^	7.79 ± 0.02^b^	0.444 ± 0.001^d^	4.86 ± 0.01^a^	11.85 ± 0.10^f^	7.54 ± .0.01^d^
V‐RWD	90	63.10 ± 1.62^e^	7.99 ± 0.489^b^	0.455 ± 0.001^c^	4.86 ± 0.01^a^	13.25 ± 0.15^c^	7.61 ± 0.05^b,c,d^
	100	36.50 ± 2.28^g^	7.89 ± 0.04^b^	0.441 ± 0.003^d^	4.85 ± 0.00^a^	12.05 ± 0.05^f^	7.69 ± 0.03^a,b^
	110	24.40 ± 0.69^h^	8.04 ± 0.40^a,b^	0.454 ± 0.003^c^	4.84 ± 0.01^a,b^	12.08 ± 0.08^e,f^	7.66 ± 0.07^b,c^
HAD	50	525.00 ± 30.00^b^	6.95 ± 0.23^c^	0.313 ± 0.001^f^	4.75 ± 0.01^e^	15.03 ± 0.03^a^	7.58 ± 0.03^b,c,d^
OD	50	930.00 ± 304.47^a^	8.29 ± 0.02^a,b^	0.418 ± 0.002^e^	4.82 ± 0.01^b,c^	12.53 ± 0.13^d^	7.79 ± 0.02^a^

Abbreviations: A‐RWD, Atmospheric Refractance Window Drying; HAD, Hot Air Drying; OD, Oven Drying; V‐RWD, Vacuum Refractance Window Drying.

^a^ Ash content calculated on a dry basis.

Values are expressed as means ± standard error. Values with different letters in the same column are significantly different (*p* < 0.05).

### 3.2 Moisture content, water activity, pH, acidity degree, and ash content

The drying process type and conditions had slight effects on the pH, acidity degree, moisture content, and water activity of tarhana samples (*p* ≤ .05) (Table 2). After drying, it was determined that the moisture content of tarhanas was 8.05 ± 0.18% and the water activity value was less than 0.6. The pH value of the dried tarhana sample varied from 4.75 to 4.86, while its acidity degree was in the range of 12.05 to 15.03. These fluctuations in pH, acidity degree, and water activity of tarhana samples may be attributed to the proportional changes due to their different moisture contents. It was reported that pH values varied from 3.5 to 5.0 (Dadalı & Elmacı, 2021; Hesseltine, 1979) and acidity degrees changed from 10 to 35 (TSE, 2004) depending on the foodstuffs used in the receipt of tarhana and process conditions. Drying processes may affect the pH and acidity degree of the tarhana sample at a limited level, as reported by Ekinci (Ekinci, 2005).

The ash content values of dried tarhanas on a dry basis varied between 7.53% and 7.79% (*p* ≤ .05). This change was thought to result from a slight homogeneity problem in tarhana dough due to local inefficient mixing, although it was aimed at having a uniform composition. The ash content of tarhanas ranged from 0.50 to 9.40%, as reported in studies (Bilgiçli, 2009; Dağdelen & Değirmencioğlu, 2005; Tamer et al., 2007).

### 3.3 Functional potential of tarhana

**FIGURE 2:**
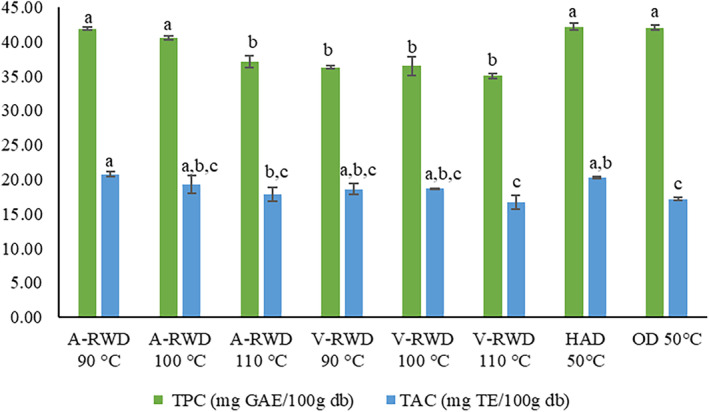
Antioxidant properties of tarhana. A‐RWD, Atmospheric Refractance Window Drying; HAD, Hot Air Drying; OD, Oven Drying; V‐RWD, Vacuum Refractance Window Drying.

TPC and TAA of tarhana samples were determined as 35.03–42.19 (mg G.A.E./100 g db) and 16.67–20.75 (mg T.E./100 g db), respectively (Figure 2). TPC and TAA of tarhana varied depending on the formulation and the drying methods. The highest TPC was achieved by the tarhana samples dried using HAD, OD, and A‐RWD at 90 and 100°C. However, the high performance of RWD in TPC was not seen in the vacuum condition, and it was found to be less than all those values corresponding to HAD, OD, and A‐RWD except for A‐RWD at 110°C. This favorable effect of A‐RWD compared to V‐RWD may be associated with the highest temperature levels the sample reached. Its corresponding value for the former one was higher than that for the latter one. The high temperatures may increase the release and extractability of matrix‐bound phenolic compounds. In coincidence with the present study, Dewanto, Wu and Liu (Dewanto et al., 2002) have also reported similar thermal process effects. Browning reactions occurring during heat treatment cause an increase in free radical scavenging capacity. This increase may be due to the decomposition of the conjugated phenolics during heat treatment, followed by some polymerization and/or oxidation reaction, and the formation of phenolics other than endogenous phenolics. Other reactions, such as the Maillard reaction, caramelization, and chemical oxidation of phenols, contribute to the increase in total phenol content (Ragaee et al., 2014). It is well known that there is a strong relation between TPC and TAA. In this study, the trends observed for both were also similar. However, unlike the TPC trend, some differences between TAA of tarhana samples obtained by different methods and/or conditions were significant (*p* ≤ .05), and some of them were not (*p* > .05) (Figure 2). The lowest TAA was obtained for tarhana samples dried by A‐RWD and V‐RWD carried at 110°C and by HAD. The remaining tarhana samples had almost identical TAA values. In this context, besides its performance on drying characteristics, tarhana production with RWD has an important potential with its high bioactive preservation effect. However, critical evaluation and optimization of temperature and pressure conditions in applications is crucial to using that potential.

### 3.4 Color values of tarhana

**FIGURE 3:**
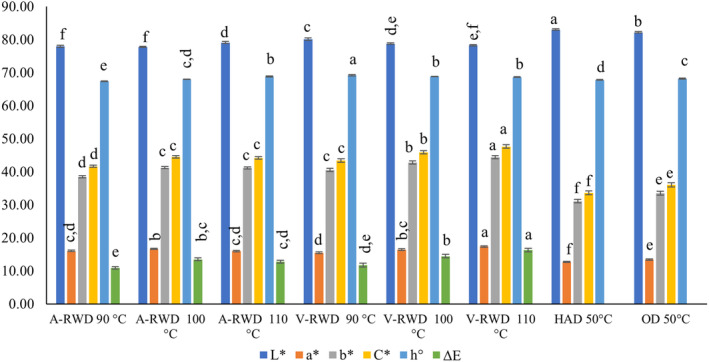
Color measurement of tarhana powders. A‐RWD, Atmospheric Refractance Window Drying; HAD, Hot Air Drying; OD, Oven Drying; V‐RWD, Vacuum Refractance Window Drying.

Lightness (L*), redness (a*), and yellowness (b*) values of dried tarhana were measured, and their corresponding values were found in the ranges of 77.78–83.02, 12.67–17.36, and 31.07–44.04. Other parameters, such as chroma (C*) and hue angle (h°) values, were calculated, and the results were given by the same equipment: 33.60–47.6 and 67.41–69.19, respectively (Figure 3). In general, the lightness of the samples decreased, and the redness increased with the browning reactions that occurred as a result of the temperature applied during the drying process. Although drying time was shorter for RWD processes, the control samples were significantly brighter and had lower redness due to lower process temperatures (50°C for OD and HAD) (*p* ≤ .05). In the drying of chickpea protein isolates (Tontul et al., 2018) and bone broth powders (Aykın‐Dinçer et al., 2021), the color parameters of samples were evaluated, and drying methods were compared. In those studies, similar to ours, it was found that RWD produced darker samples with higher redness values than freeze‐dried ones. The same effect on the color of goldenberry pulp was reported by Puente‐Díaz, Spolmann, Nocetti, Zura‐Bravo, and Lemus‐Mondaca (Puente‐Díaz et al., 2020) in which RWD was compared with different drying methods (freeze‐drying [FD], convective drying [CD], and infrared drying [IRD]). In the current study, similar to the redness values, the measured yellowness and chroma values of the RWD samples were significantly higher than the control groups (OD and HAD) had (*p* ≤ .05). Additionally, in the RWD method, the increase in temperature caused significant increases in the redness and yellowness values when the process temperature shifted from 90°C to 100°C (*p* ≤ .05), but further increase did not create any more effect (*p* > .05). However, increases in redness and yellowness values of tarhana samples dried by RWD were observed for all studied temperature levels when the process was carried out under vacuum (*p* ≤ .05) (Figure 3). A clear trend could not be determined for the Hue angle value of tarhana, but the highest h° value was determined for V‐RWD 90°C and the lowest for A‐RWD 90°C.

The color values of the conventionally dried (commonly used in the food industry) samples at 50°C were used to calculate the color change in the dried tarhanas. The shift in color values caused by RWD is the highest in V‐RWD/110°C and the lowest in A‐RWD/90°C dried samples.

### 3.5 Techno‐physical properties of tarhana powders

Tarhana contains milk proteins (casein, globulin, and albumin), wheat proteins (glutenin and gliadin), and starch due to the yogurt and flour used in its formulation (Çelik et al., 2005). Solubility, water and oil holding capacity, foaming capacity (FC), stability, flowability, and cohesiveness are critical techno‐physical properties of tarhana powder, and they affect product quality mainly due to its protein and starch contents.

Solubility is an important quality criterion for soups that are prepared with water. The solubility values of tarhana samples produced by the RWD technique at different studied conditions were higher than those dried by HAD and OD (Table 3). Although it is well known that solubility is significantly reduced by denaturation of proteins at elevated temperature levels like 110°C under both atmospheric and vacuum conditions, the solubilities of corresponding samples are still higher than the control groups' (*p* ≤ .05) (Table 3). The literature reported that an increase in the drying temperature adversely affected the solubility values of tomato powder and decreased it (Abul‐Fadl & Ghanem, 2011). In another study, the results indicated that the solubility of kefir powder was not changed with a temperature increase during the drying process (Tontul et al., 2021). The porosity of tarhana powder (38.39%–41.03%) was also investigated, and no significant change was seen depending on drying methods and process parameters (*p* > .05) (Table 3). Tarhana is not hygroscopic and can be stored for 1–2 years (İbanoğlu & İbanoğlu, 1999). However, the extent of this effect in tarhana depends on the drying method used and the final moisture content. Therefore, these differences in hygroscopicity values are due to the different final moisture contents of the product.

**TABLE 3:** Techno‐physical properties of tarhana powders.

Drying process	Temperature, °C	Solubility^a^ (%)	Porosity (*Ɛ*) (%)	Flowability (CI)	Cohesiveness (HO)	Hygroscopicity (%)	Foam capacity (mL/mL)	Foam stability (%)	Water holding capacity^a^	Oil holding capacity^a^
A‐RWD	90	84.11 ± 0.05^a^	41.03 ± 2.58^a^	28.65 ± 1.88^c,d^	1.29 ± 0.02^c,d^	10.58 ± 0.65^d^	0.115 ± 0.01^a,b,c^	71.67 ± 6.73^a^	1.63 ± 0.00^a^	1.07 ± 0.02^a^
	100	83.84 ± 0.11^a,b^	40.81 ± 1.56^a^	27.98 ± 1.97^c,d^	1.28 ± 0.02^c,d^	11.16 ± 0.21^c,d^	0.104 ± 0.01^a,b,c,d^	66.67 ± 9.61^a^	1.50 ± 0.00^b,c^	1.05 ± 0.01^a,b^
	110	83.42 ± 0.08^c^	39.40 ± 2.11^a^	25.00 ± 0.00^d^	1.25 ± 0.00^d^	11.89 ± 0.47^b,c,d^	0.092 ± 0.03^b,c,d^	29.44 ± 10.82^b,c^	1.27 ± 0.00^d^	1.01 ± 0.01^b^
V‐RWD	90	83.54 ± 0.17^b,c^	40.75 ± 1.63^a^	30.97 ± 1.68^b,c^	1.31 ± 0.02^b,c^	11.46 ± 0.04^c,d^	0.093 ± 0.01^b,c,d^	27.78 ± 2.77^b,c^	1.57 ± 0.00^a,b^	1.08 ± 0.01^a^
	100	83.87 ± 0.07^a,b^	40.01 ± 2.25^a^	30.08 ± 0.90^c,d^	1.30 ± 0.01^c,d^	12.97 ± 0.37^a,b^	0.147 ± 0.01^a,b^	33.33 ± 9.61^b^	1.42 ± 0.00^c^	1.08 ± 0.02^a^
	110	83.45 ± 0.12^c^	39.32 ± 0.43^a^	26.90 ± 0.00^c,d^	1.27 ± 0.00^c,d^	12.96 ± 0.70^a,b^	0.161 ± .0.03^a^	36.67 ± 1.93^b^	1.27 ± 0.04^d^	1.11 ± 0.00^a^
HAD	50	83.09 ± 0.07^d^	38.61 ± 2.88^a^	38.77 ± 3.10^a^	1.39 ± 0.03^a^	13.84 ± 0.13^a^	0.071 ± 0.00^c,d^	10.33 ± 0.00^c^	0.79 ± 0.08^e^	0.90 ± 0.02^c^
OD	50	82.65 ± 0.10^e^	38.39 ± 2.48^a^	35.38 ± 1.18^a,b^	1.36 ± 0.01^a,b^	12.41 ± 0.11^b,c^	0.052 ± 0.00^d^	13.78 ± 0.00^b,c^	0.72 ± 0.00^e^	0.92 ± 0.03^c^

Abbreviations: A‐RWD, Atmospheric Refractance Window Drying; HAD, Hot Air Drying; OD, Oven Drying; V‐RWD, Vacuum Refractance Window Drying.

^a^ Solubility, WHC, and OHC calculated on a dry basis.

Values are expressed as means ± standard error. Values with different letters in the same column are significantly different (p < 0.05).

A literature survey revealed that desired or undesired changes in the foaming properties of cereal products may occur depending on the heat treatment (Chavan et al., 1989). The foaming properties, FC, and foam stability (FS) of tarhana samples were examined in the current study. FC and FS values varied depending on the drying methods and the applied process parameters (*p* ≤ .05) (Table 3). As can be seen in Table 3, the highest FC was for tarhana samples dried by A‐RWD at 90°C and 100°C and by V‐RWD at 100°C and 110°C. The lowest values were determined in the samples obtained using A‐RWD at 110°C, V‐RWD at 90°C, HAD at 50°C and OD at 50°C. Similar to the FC of samples, tarhana powders produced using A‐RWD 90°C and A‐RWD 100°C showed the highest FS values (Table 3). On the other hand, the foams produced by tarhana samples dried by A‐RWD at 110°C, by V‐RWD at 90°C, by HAD at 50°C, and by OD at 50°C had the lowest stability (Table 3). When the RWD was evaluated in terms of process parameters, temperature increases negatively affected FS (*p* ≤ .05) under atmospheric pressure. In contrast, temperature change in the RWD process under vacuum did not cause any significant change in FS (*p* > .05) (Table 3). The differences between the FC and FS values of the dried powder tarhana samples may be attributed to the resulting structural changes depending on the protein denaturation due to the heat treatment applied during drying and its duration in RWD, where temperature raised drastically, especially under atmospheric conditions. On the other hand, although the temperature was low in HAD and OD, the long processing times became effective on the foam capacity and stability, and it negatively changed them (*p* ≤ .05). In a study examining the effect of heat treatment on the foam properties of tarhana, it was reported that the temperature decreased the foam capacity and stability (İbanoğlu & İbanoğlu, 1997).

WHC, another important techno‐physical property, was also examined in this study. The WHC of tarhana samples changed from 1.27 to 1.63 g/g db for RWD processes (Table 3). Similar to this study, the WHC of tarhana was studied, and its change depending on drying conditions was examined by Hayta, Alpaslan and Baysar (Hayta et al., 2002). The WHC results (0.45–2.28 g/g) coincided with ours. (Hayta et al., 2002). When different drying methods were compared, it was seen that the WHC of the RWD samples was significantly higher than that of tarhana samples processed by HAD and OD (*p* ≤ .05) (Table 3). WHC is one of the gelatinization indexes (Yaşacan, 2002). The gelatinization temperature of wheat starch is 58–65°C (Yüksel, 2020). The drying temperature in the RWD method was in the range of 90–110°C. For the RWD processes under these conditions, the highest temperature level of the drying sample was around 85°C. This value was well above the gelatinization temperature. Thus, it can be concluded that the WHC of samples dried with RWD was higher for both pressure conditions under vacuum and atmosphere compared to HAD and OD since starch gelled during RWD, whereas the reached highest temperature level was not enough for starch gelatinization in the control processes. On the other hand, the process temperature for RWD adversely affected the WHC of tarhana samples. This can be associated with the adverse effect of temperature on protein structure. The decrease in WHC with increasing drying temperature is due to the increase in damage to the structure of proteins. Because WHC is directly related to the structural state of the proteins (Deng et al., 2002). However, only considering temperature as an effective parameter is not a proper approach since the temperature effect should need to be considered with acting time to assess outcomes adequately. In this study, although RWD were at elevated temperature levels compared to HAD and OD, total process time was not longer than those taken in HAD or OD. Thus, the adverse effects of the high temperature of RWD remained limited.

Oil holding capacity (OHC) is defined as the amount of oil absorbed by the food matrix (Hayta et al., 2002). In general, flavor and odor substances of food materials are dissolved in oil, so the OHC of the product is an important quality criterion regarding sensorial properties. The hydrophilic character of starch, fiber content, total load density, surface properties, and hydrophobic properties of the proteins all affect OHC (Alkarkhi et al., 2011; Borchani et al., 2011; Hayta et al., 2002). The OHC of tarhana dried with RWD was in the 0.90–1.11 g/g db range. These results were higher than the literature reported by Hayta, Alpaslan, and Baysar (Hayta et al., 2002). This can be due to different drying techniques and/or differences in the raw materials used in the formulation. The OHC values of RWD samples were significantly higher than control groups (*p* ≤ .05).

Flowability and cohesiveness are two important techno‐physical properties of powder products. Flowability and cohesiveness of tarhana powders were calculated by the Carr index (CI) and Hausner ratio (HR), respectively. CI values of tarhana powders produced by RWD significantly differed from the control groups' (*p* ≤ .05) (Table 3). Lower CI and HR values are suitable for better processing of powders. The Carr index of tarhana produced with RWD was 25.00–30.08, which was evaluated as fair flow, as the control groups were above 35, indicating bad flow characteristics. Hausner ratios of tarhanas were between 1.2 and 1.4, and samples in that range were classified as intermediately cohesive. Similar to the flowability properties, the highest cohesiveness values were determined for samples obtained by HAD and OD, and the lowest was found for tarhana powder when A‐RWD processed it at 110°C. In general, RWD's process parameters did not create a clear difference in the flowability and cohesiveness properties of tarhana powder (*p* > .05). In other words, temperature change and pressure conditions were not significant factors. On the other hand, the drying method changed flowability and cohesiveness (*p* ≤ .05). The methods used for control groups, especially HAD, differed from RWD. According to expectations regarding flow characteristics, RWD resulted in tarhana samples with better flowability and cohesiveness. Tontul, Ergin, Eroğlu, Küçükçetin, and Topuz (Tontul et al., 2021) studied kefir powders produced with RWD and reported that the samples had good flowability and low cohesiveness. Nansereko, Muyonga, and Byaruhanga (Nansereko et al., 2022) investigated the effects of the RWD method on the techno‐physical properties of dried jackfruit powders, and it was found that the powder flowability of samples obtained by this method was superior to that of freeze‐dried powder. The flowability of samples dried by RWD was classified as excellent, whereas freeze‐dried powder exhibited good flowability.

### 3.6 Rheological properties of tarhana

The rheological properties of soups during food processing provide insight into the structure of the components in the product. In addition, the rheological properties, an important sensory quality characteristic of flowing products such as soups, also affect consumer preference. The variation of the shear stress data corresponds to the shear rate for soups prepared by tarhana–water mixtures from the samples dried with different drying techniques. This was explained with the commonly used rheological models, Newtonian, Power Law, Herschel–Bulkey, and Casson models. The estimated model parameters are given in Table 4. According to model performance parameters, it is clear that the soup samples did not display Newtonian behavior since the Newtonian model showed poor performance (*R* ^2^ < .90 for most of the samples) to explain the variation in shear stress value as a function of shear rate (Table 4). When non‐Newtonian model performances were evaluated, the Herschel–Bulkey model was one step ahead of others, especially for temperature levels of 70°C and 55°C attained during cooling. The flow behavior of soup samples can be assessed by considering the flow behavior constant (n). When it is shifted from 1, it means the flow is not like a Newtonian fluid does (Al‐Malah et al., 2000). As can be seen from Table 4, the n value of the dried tarhana sample was less than one and exhibited pseudoplastic (shear‐thinning) flow behavior so that it can be classified as a non‐Newtonian fluid (İbanoğlu & İbanoğlu, 1999), similarly to Hayta, Alpaslan, and Baysar (Hayta et al., 2002). The consistency coefficient value (k) also gives information about the viscosity of the samples (Yilmaz et al., 2010). The increase in k value in fluids indicates that it is more viscous, or, in other words, its viscosity is higher. The viscosity value for all soups prepared using dried tarhana samples decreased with cooling from 70°C to 40°C. On the other hand, drying methods affected the viscosity value of soups. Comparing the consistency index, it can be concluded that viscosity values calculated for tarhana samples dried by RWD were higher than those determined for control groups. That may be attributed to the pre‐gelatinization of starch granules in tarhana dough due to the temperature level of drying attained in the RWD system. As the temperature level cannot exceed 50°C for control group drying, the starch gelatinization temperature level has already passed for drying tarhana samples in the RWD system, irrespective of the pressure condition applied.

**TABLE 4:** Rheological model parameters describing the flow behavior indices of tarhanas at different temperatures.

	Temperature, °C	Newtonian model			Power law model				Herschel–Bulkey model					Casson model			
		*k*	RMSE	*R* ^2^	*n*	*k*	RMSE	*R* ^2^	*n*	*k*	T_0_	RMSE	*R* ^2^	*k*	T_0_	RMSE	*R* ^2^
OD 50°C	40	1.148	14.972	.702	0.206	16.480	2.888	.833	0.206	16.480	0.000	2.917	.833	0.092	16.581	4.004	.679
	55	1.133	14.319	.714	0.207	16.147	2.013	.906	0.207	16.147	0.000	2.032	.906	0.093	16.119	3.087	.778
	70	1.029	9.817	.815	0.304	10.565	0.901	.984	0.405	6.208	5.119	0.819	.987	0.173	10.029	1.212	.972
HAD 50°C	40	1.145	14.729	.707	0.212	16.118	2.834	.843	0.212	16.118	0.000	2.862	.843	0.097	16.180	3.961	.694
	55	0.833	6.979	.851	0.353	7.279	0.612	.991	0.530	3.123	5.283	0.242	.999	0.179	6.732	0.481	.994
	70	0.545	4.124	.875	0.398	4.111	0.389	.993	0.538	2.132	2.625	0.221	.998	0.141	3.712	0.404	.992
A‐RWD 90°C	40	1.104	12.481	.758	0.252	13.533	1.656	.947	0.252	13.533	0.000	1.672	.947	0.132	13.262	2.720	.857
	55	0.876	7.262	.853	0.361	7.468	0.353	.997	0.455	4.692	3.447	0.132	1.000	0.195	6.878	0.712	.989
	70	0.489	3.743	.872	0.389	3.795	0.464	.986	0.615	1.350	3.318	0.153	.999	0.122	3.444	0.203	.997
A‐RWD 100°C	40	0.177	1.166	.903	0.452	1.122	0.168	.989	0.657	0.452	0.985	0.066	.998	0.055	0.975	0.077	.998
	55	0.131	0.706	.932	0.528	0.644	0.108	.993	0.690	0.319	0.527	0.046	.999	0.051	0.517	0.057	.998
	70	0.090	0.371	.959	0.621	0.326	0.043	.998	0.701	0.232	0.171	0.022	.999	0.045	0.221	0.044	.998
A‐RWD 110°C	40	0.635	5.007	.865	0.380	5.079	0.322	.996	0.504	2.815	2.911	0.060	1.000	0.153	4.626	0.424	.993
	55	0.492	4.087	.853	0.355	4.271	0.409	.988	0.570	1.546	3.522	0.083	1.000	0.106	3.944	0.189	.997
	70	0.332	2.607	.966	0.376	2.684	0.384	.979	0.669	0.714	2.697	0.100	.999	0.079	2.454	0.102	.998
V‐RWD 90°C	40	0.059	0.198	.973	0.681	0.174	0.019	.999	0.716	0.150	0.047	0.016	.999	0.034	0.099	0.035	.997
	55	0.049	0.180	.968	0.657	0.158	0.019	.999	0.704	0.129	0.054	0.015	.999	0.027	0.097	0.030	.997
	70	0.039	0.117	.978	0.712	0.105	0.013	.999	0.737	0.094	0.022	0.012	.999	0.024	0.053	0.023	.998
V‐RWD 100°C	40	1.033	9.116	.837	0.351	9.151	0.940	.987	0.351	9.151	0.000	0.949	.987	0.218	8.515	1.910	.946
	55	0.845	6.837	.860	0.371	6.961	0.314	.998	0.460	4.512	3.072	0.082	1.000	0.196	6.374	0.695	.989
	70	0.639	5.381	.850	0.350	5.633	0.507	.989	0.548	2.196	4.391	0.152	.999	0.135	5.221	0.324	.996
V‐RWD 110°C	40	1.043	9.591	.826	0.334	9.755	1.002	.984	0.334	9.755	0.000	1.012	.984	0.203	9.166	2.009	.937
	55	0.843	6.871	.857	0.368	7.017	0.300	.998	0.454	4.595	3.021	0.068	1.000	0.193	6.436	0.699	.989
	70	0.568	4.680	.855	0.357	4.880	0.556	.984	0.599	1.584	4.319	0.193	.998	0.124	4.506	0.252	.997

Abbreviations: A‐RWD, Atmospheric Refractance Window Drying; HAD, Hot Air Drying; OD, Oven Drying; V‐RWD, Vacuum Refractance Window Drying.

Lower viscosity values at higher temperature levels can be associated with the destabilization that occurs in protein–protein and protein–water interactions with increasing temperature and leads to a decrease in viscosity (Huang & Kinsella, 1986).

### 3.7 Scanning electron microscopy (SEM) imaging of dried tarhana

**FIGURE 4:**
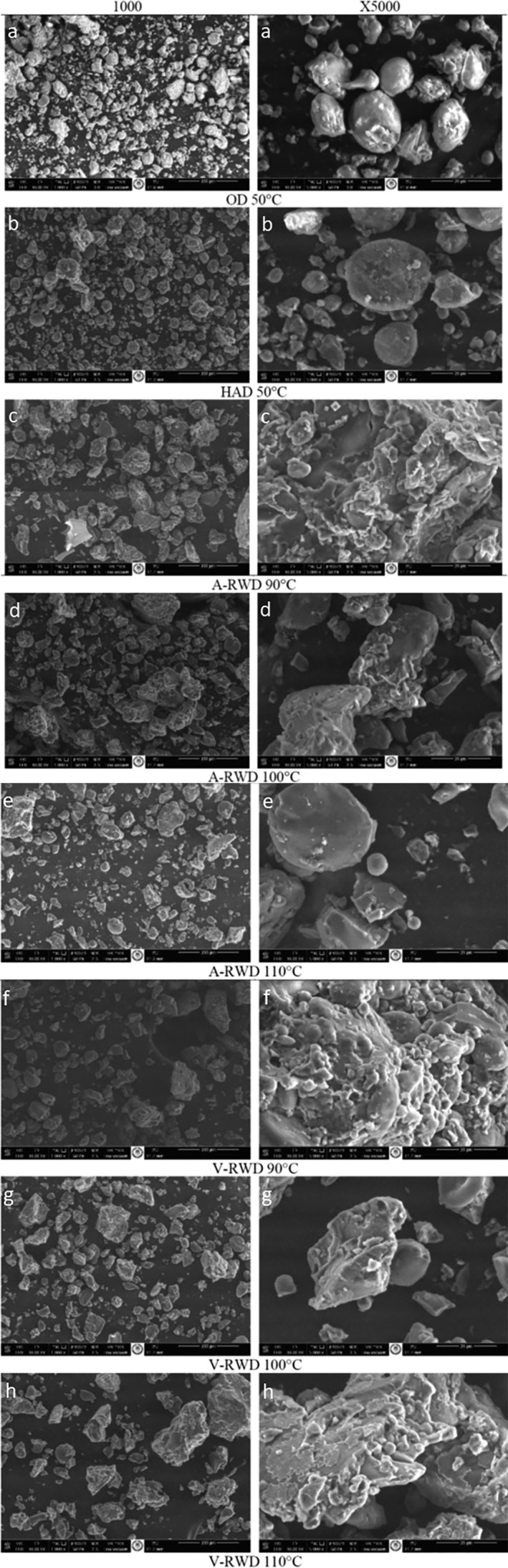
Scanning electron microscopy images of tarhana powders. A‐RWD, Atmospheric Refractance Window Drying; HAD, Hot Air Drying; OD, Oven Drying; V‐RWD, Vacuum Refractance Window Drying.

SEM images of tarhana powders were displayed in Figures 4. Ungelatinized starch is in the form of oval granules (Yaşacan, 2002). On the other hand, gelatinized starch granules are observed to have a layer‐like structure (Majzoobi et al., 2011). In the current study, oval starch granules were seen on the SEM images of the control groups (Figure 4a,b). Drying processes for control groups of tarhana samples were performed at 50°C, which was thought not high enough for complete starch gelatinization. Thus, observing oval forms of starch granules coincides with the results reported by Yaşacan (Yaşacan, 2002). The RWD process causes samples temperature to exceed starch gelatinization level in the current study, so similar to the observations by Majzoobi, Radi, Farahnaky, Jamalian, Tongtang, and Mesbahi (Majzoobi et al., 2011), layer‐like structure was also visualized in those tarhana samples (Figure 4c–h). It can be said that the samples dried with RWD form a more irregular and intermittent or protruding image. The temperatures applied in both RWD methods are above the temperature required for starch gelatinization. On the other hand, when tarhana samples dried by RWD under atmospheric and vacuum conditions were compared to each other concerning micro‐structure visualized in SEM images, it can be said that a similar final structural form was obtained in both drying conditions. Furthermore, some particles were seen to be covered with a thin layer on SEM images. Studies have reported that these were fat layers (Do et al., 2011; Salameh et al., 2016). However, those layers were not observed on all images or all particles in one image. This is because tarhanas do not have a high fat content (Goencue & Celik, 2020).

## 4 CONCLUSION

The current study serves the literature by providing new information about tarhana production with the RWD technique and the effects of drying methods on tarhana under atmospheric and vacuum conditions. Considering the results, it can be revealed that the process time savings were over 85% and 75% compared to OD and HAD, respectively. Additionally, the pressure condition of the RWD was also critical. Tarhana was dried under vacuum for approximately 50% less than the drying time required under atmospheric conditions. In drying processes, losses occur in bioactive compounds due to temperature–time relations. Despite the high temperature levels reached in RWD, this technique provided high bioactive compound preservation, and the results were comparable to the control groups. It was found that the techno‐physical properties of tarhana (flowability, stickiness, solubility, water and oil holding capacity, foam capacity, and stability) dried by RWD were better than control samples. Based on this, it can be argued that the RWD system has significant potential for the food industry to produce tarhana both under atmospheric and vacuum conditions. It was concluded that the system's efficiency has improved with the vacuum application adapted to the RWD system. Still, it requires more studies at different pressure levels for other materials in this field.

## AUTHOR CONTRIBUTIONS

**Merve Seçil Bardakçi:** Conceptualization (equal); data curation (equal); formal analysis (equal); investigation (equal); methodology (equal); validation (equal); writing – original draft (equal); writing – review and editing (equal). **Erkan Karacabey:** Conceptualization (equal); data curation (equal); formal analysis (equal); investigation (equal); methodology (equal); project administration (lead); resources (equal); supervision (equal); validation (equal); visualization (equal); writing – original draft (equal); writing – review and editing (equal).

## FUNDING INFORMATION

The publication fee of the study is supported by TUBITAK (TUB1).

## CONFLICT OF INTEREST STATEMENT

The authors declare no conflict of interest.

## Data Availability

The data presented in this study are available on request from the corresponding author.
